# Sense of agency strengthens memory for self-caused spatial information

**DOI:** 10.1016/j.isci.2026.115382

**Published:** 2026-03-16

**Authors:** Qiaoyue Ren, Bruno Herbelin, Nathalie H. Meyer, Sara Stampacchia, Simone Schütz-Bosbach, Olaf Blanke

**Affiliations:** 1 Laboratory of Cognitive Neuroscience, Neuro-X Institute, Faculty of Life Sciences, Ecole Polytechnique Fédérale de Lausanne, Geneva, Switzerland; 2 Department of Intelligent Precision Healthcare Convergence, Sungkyunkwan University, Suwon, Republic of Korea; 3 General and Experimental Psychology Unit, Department of Psychology, LMU Munich, Munich, Germany

**Keywords:** health sciences, clinical neuroscience, psychology

## Abstract

Why do some experiences remain vivid while others fade? One factor may be the sense of agency (SoA), which shares self-referential mechanisms with episodic memory. We investigated whether SoA over action outcomes modulates item and spatial long-term memory. During intentional encoding, participants’ keypresses moved an object either congruently or incongruently with the chosen direction. One hour later, item and spatial memory were tested. Participants reported higher SoA in congruent trials. Congruency and higher SoA ratings were associated with faster item-recognition responses without changes in sensitivity, suggesting enhanced decision efficiency rather than improved item-memory quality. In contrast, both predicted higher spatial memory accuracy, independent of encoding duration, spatial bias, or guessing. These findings indicate that perceived control during encoding selectively strengthens memory for the spatial consequences of one’s actions, supporting a role for SoA in binding actions and outcomes into episodic representations.

## Highlights

• Action-outcome congruency and stronger agency at encoding sped item recognition
• Congruency and stronger agency enhanced memory for spatial action outcomes
• Sense of agency supports binding actions and outcomes into episodic memory

## Graphical abstract

**Figure:**
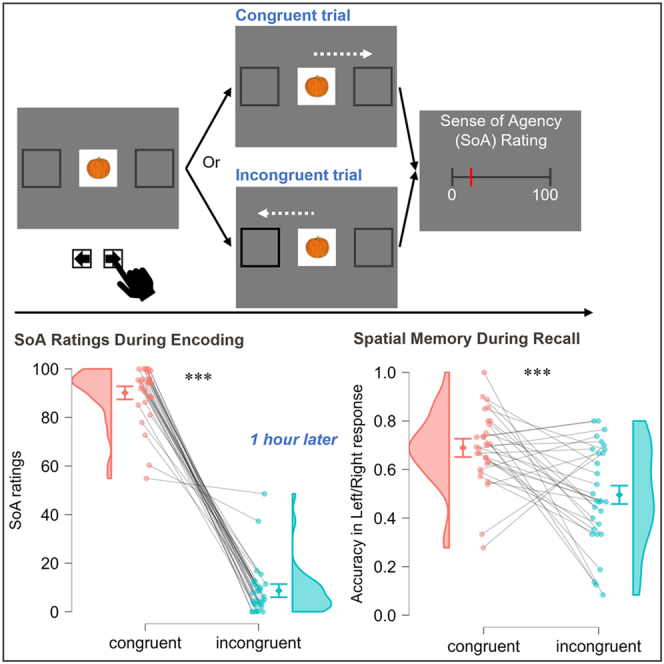

## Introduction

Episodic memory (EM), the ability to consciously recollect personally experienced events in rich detail,^1^^,^^2^ is a cornerstone of human cognition. It links past events to our sense of self and identity, and shapes decision-making, future planning, and social interactions.^3^^,^^4^ Yet, why are some past experiences vividly remembered while others fade? While factors such as emotional salience^5^ and sensory richness^6^ are well-established contributors to EM, the role of the sense of agency (SoA)—the feeling of controlling one’s own actions and the effects of these actions on elements of the environment^7^—has been largely overlooked.

Remembering a past event involves not only retrieving individual details but also reconstructing causal relationships among event elements,^8^ such as attributing event details as related to one’s own actions or to external causes. This attribution process parallels the core mechanism of SoA, i.e., distinguishing between self-caused and externally driven changes in the environment.^7^ For example, when recalling placing newly purchased items into specific drawers at home yesterday, one readily identifies oneself, not another person, as the agent of the action (placing the item) and the outcome (the item’s stored location). A heightened SoA during an event may enhance event encoding by prioritizing such self-relevant information.^9^ Additionally, motor interactions with the environment that evoke strong SoA have been shown to be intrinsically motivating,^10^^,^^11^ which may foster deeper engagement and more elaborate consolidation processes, thereby leading to stronger and more durable memory traces.

Prior research provides indirect support for a facilitation effect of SoA on memory. For example, physically performing actions represented by words (e.g., “clap your hands”) leads to better memory of these words than simply reading them.^12^ Similarly, self-directed learning, where individuals control the timing and sequence of study materials, yields memory advantages.^13^^,^^14^^,^^15^ Even simple key-press actions in a Go/No-Go task can boost memory.^16^ Moreover, participants show stronger memory when making free action choices rather than forced ones,^17^^,^^18^^,^^19^ and when actively controlling navigation in a virtual city compared to passively observing.^20^ These findings suggest a possible link between SoA and memory, as SoA is typically stronger during active and freely chosen actions.^21^^,^^22^ However, most of these studies did not directly measure SoA, and the reported effects may reflect, at least in part, confounding factors such as increased motor engagement during active control versus passive observeration^16^ or greater opportunities for selective restudy of previously unremembered items in self-directed versus other-determined learning sequences,^23^ rather than SoA per se.

Although some recent studies have directly measured SoA, these investigations have primarily focused on working or short-term memory for simple stimuli, rather than EM, which involves long-term retention and the recall of rich event details (e.g., temporal or spatial elements). For instance, Zou and colleagues^24^ showed that SoA can enhance working memory. In their study, participants moved several boxes on a screen with arrow keys, one of which differed in controllability. Afterward, an icon (black-and-white line drawings of objects) appeared inside each box for encoding, followed by a recognition test for one of the items and SoA rating. Items associated with higher SoA were remembered better, suggesting that SoA enhances working memory. Other studies found that congruency between participants’ keypresses and the movement of words on a screen enhanced both SoA ratings during encoding and recognition accuracy for those words tested immediately after encoding.^25^^,^^26^^,^^27^ However, such SoA effects on short-term memory are inconsistent, with another study using similar paradigms but finding no memory benefits despite successful SoA modulation.^28^ Importantly, all of these findings are restricted to immediate recognition of individual items, leaving open the question of whether SoA influences long-term EM for richer event details.

**Figure 1:**
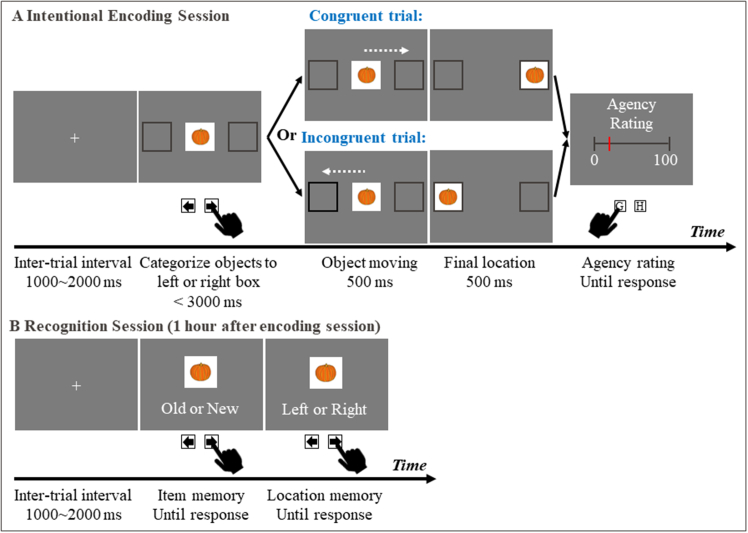
Schema of the agency-memory task (A) Encoding session, during which participants experienced either congruent or incongruent object movement and intentionally encoded both the item and its final location. (B) Recognition session, during which participants’ memory for the items and their spatial locations was tested.

Here, we aimed to investigate whether SoA over object displacement influences EM for these objects and their spatial locations. During encoding, participants (*N* = 30) categorized objects by moving them into a left or right box via keypresses, while we manipulated the congruency between the chosen action and the ensuing movement direction of the objects on the screen (see Figure 1A). This manipulation is known to modulate explicit judgments of agency.^26^^,^^27^^,^^29^^,^^30^ Participants reported their experienced SoA on each trial, enabling both validation of the manipulation and trial-by-trial analyses of the relationship between SoA and EM. In contrast to prior work that assessed memory immediately after encoding and thus primarily captured short-term memory processes, the present study assessed memory after a 1-h delay to target long-term EM. At test, participants judged whether objects were old or new and, for previously seen objects, recalled their final spatial location (see Figure 1B). We found that action-outcome congruency and the resulting enhancement in SoA during encoding selectively strengthened subsequent memory for spatial action outcomes, whereas effects on item recognition were expressed primarily in faster response times (RTs) rather than improved old-new discrimination.

## Results

### Congruent action-outcome mappings increased SoA

**Figure 2:**
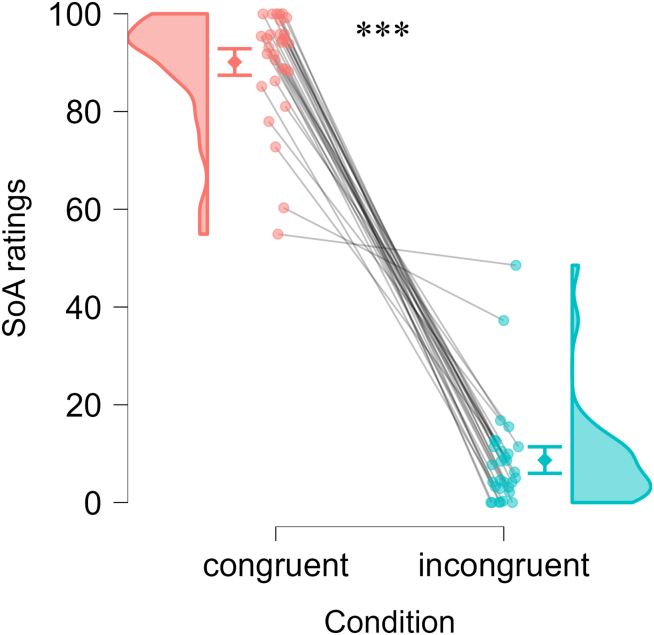
Sense of agency (SoA) ratings Dots show individual participants (connected across the two conditions; *N* = 30). Diamonds represent group means, with error bars showing the standard error of the paired difference. ∗∗∗: *p* < 0.001 by paired-samples *t* test.

A paired-samples *t* test confirmed the effectiveness of the manipulation of the SoA: During the encoding session, participants reported significantly higher SoA ratings in the congruent condition (i.e., pressing “left” key moved the object to the left; M = 90.14, SD = 11.12) compared to the incongruent condition (i.e., pressing “left” key moved the object to the right; M = 8.69, SD = 10.59), *t*(29) = 21.21, *p* < 0.001, Cohen’s *d* = 3.87 (see Figure 2).

### Congruency and higher SoA facilitated faster item recognition without affecting old-new discrimination

To assess item memory, we examined participants’ performance in the old/new recognition task, focusing on both recognition sensitivity (old-new discrimination) and RTs. We separately examined the effects of (1) objective task conditions (congruent versus incongruent), serving as an indirect proxy of SoA, and (2) subjective agency experience (SoA ratings), serving as a direct measure of SoA, while controlling for encoding duration (RTs during the encoding session). The two predictors were modeled separately to avoid collinearity, given that SoA ratings were directly influenced by the congruency manipulation.

#### Recognition sensitivity in old/new response

The first generalized linear mixed-effects models (GLMMs) included fixed effects of congruency and encoding duration, with random intercepts for participant and image. Congruency was not a significant predictor of recognition sensitivity (β = −0.06, SE = 0.12, z = −0.52, *p* = 0.606). In contrast, encoding duration significantly predicted recognition sensitivity (β = 0.24, SE = 0.07, z = 3.58, *p* < 0.001), such that longer encoding durations were associated with a higher likelihood of correctly recognizing old items.

The second GLMM included SoA ratings and encoding duration as fixed effects, with the same random effects structure. SoA ratings did not significantly predict recognition sensitivity (*β* = −0.03, SE = 0.06, z = −0.41, *p* = 0.680), whereas encoding duration again showed a significant positive effect (β = 0.24, SE = 0.07, z = 3.58, *p* < 0.001).

In summary, these results suggest that encoding duration, rather than the congruency manipulation or the related changes in SoA ratings, was the primary factor influencing item recognition sensitivity, that is, participants’ ability to distinguish old items from new items.

#### RTs in old/new response

**Figure 3:**
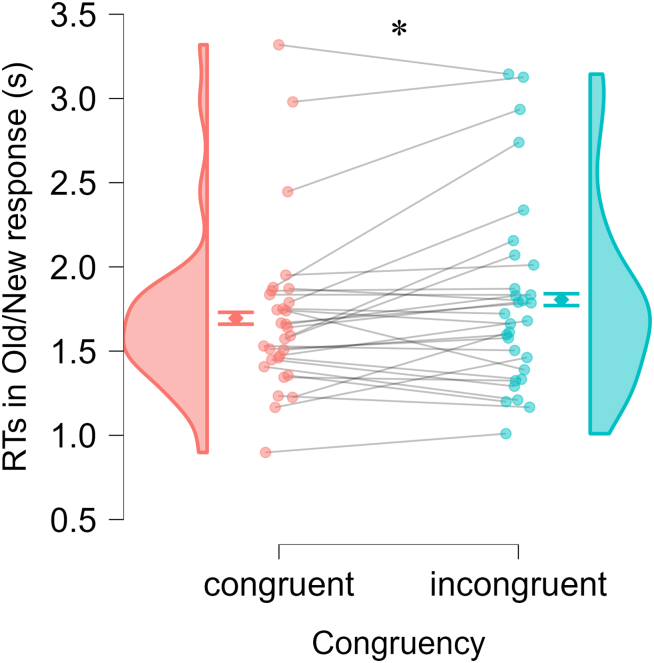
Reaction times (RTs) for old images in old/new response Dots show individual participants (connected across the two conditions; *N* = 30). Diamonds represent group means, with error bars showing the standard error of the paired difference. ∗: *p* < 0.05 by linear mixed-effects model.

As recognition sensitivity was not significantly affected by either congruency or SoA ratings, RT analyses were first conducted on all trials regardless of accuracy. The first linear mixed-effects model (LMM) included fixed effects of congruency and encoding duration, with random intercepts for participant and image. Congruency significantly predicted RTs (β = −0.11, SE = 0.05, *t* = −2.26, *p* = 0.024), suggesting that the congruent condition led to faster old/new responses compared to the incongruent condition (see Figure 3). Encoding duration did not significantly predict RTs (β = 0.01, SE = 0.02, *t* = 0.21, *p* = 0.830).

The second LMM included SoA ratings and encoding duration as fixed effects, with the same random effects structure. SoA ratings were a significant predictor, with higher SoA ratings associated with faster old/new responses (β = −0.05, SE = 0.02, *t* = −2.08, *p* = 0.037), whereas encoding duration again did not significantly predict RTs (β = 0.01, SE = 0.02, *t* = 0.23, *p* = 0.821).

To explore whether these effects depended on successful recognition, additional exploratory LMMs were run on trials with correct old/new responses only. In these models, none of the predictors significantly predicted RTs (model 1: congruency: β = −0.07, SE = 0.06, *t* = −1.18, *p* = 0.239; encoding duration: β = −0.01, SE = 0.03, *t* = −0.35, *p* = 0.727; model 2: SoA ratings: β = −0.03, SE = 0.03, *t* = −1.08, *p* = 0.279; encoding duration: β = −0.01, SE = 0.03, *t* = −0.35, *p* = 0.728).

In summary, these results suggest that the congruency manipulation and the related changes in SoA ratings, but not encoding duration, modulated the speed of old/new recognition responses. However, these effects were observed only when including all trials and did not hold when analyses were restricted to correct responses, indicating an influence on decision-related processes rather than on item memory quality per se.

### Congruency and higher SoA ratings enhanced spatial memory accuracy without changes in response speed

For spatial memory, we examined accuracy and RTs in the left/right response for correctly recognized old images, using mixed-effects models similar to item memory analysis.

#### Accuracy in left/right response

**Figure 4:**
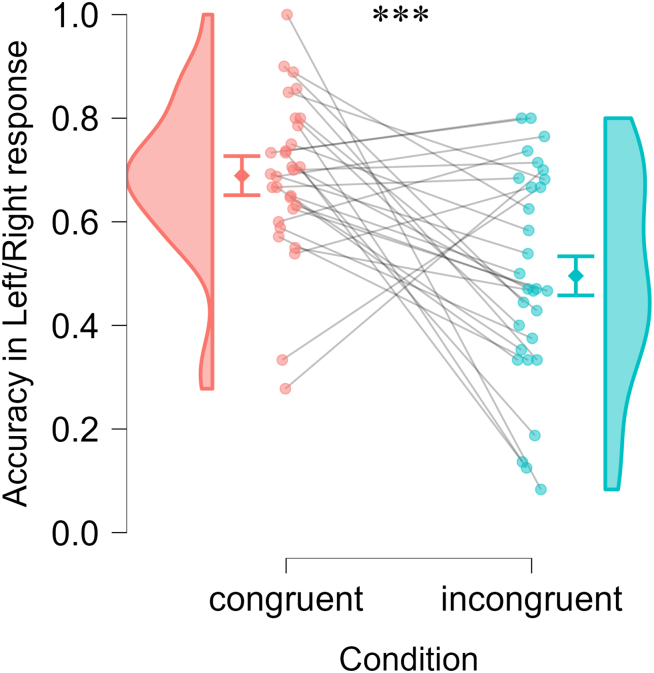
Accuracy in the left/right response for correctly recognized old images Dots show individual participants (connected across the two conditions; *N* = 30). Diamonds represent group means, with error bars showing the standard error of the paired difference. ∗∗∗: *p* < 0.001 by generalized linear mixed-effects model.

The first GLMM included fixed effects of congruency and encoding duration, with random intercepts for participant and image. Congruency was a significant predictor of spatial accuracy (β = 0.40, SE = 0.07, z = 5.80, *p* < 0.001), with higher accuracy in the left/right response in the congruent compared to the incongruent condition (see Figure 4). Encoding duration was not a significant predictor (β = −0.02, SE = 0.07, z = −0.28, *p* = 0.780).

The second GLMM included SoA ratings and encoding duration as fixed effects, with the same random effects structure. SoA ratings were a significant predictor (β = 0.32, SE = 0.07, z = 4.70, *p* < 0.001), such that stronger SoA during encoding was associated with higher accuracy in the left/right response. Encoding duration was again not significant (β = −0.02, SE = 0.07, z = −0.29, *p* = 0.772). Following a reviewer’s suggestion, we additionally examined whether SoA ratings predicted spatial memory accuracy within each condition (congruent and incongruent), using GLMMs. Neither SoA ratings nor encoding duration significantly predict spatial memory accuracy in the congruent condition (SoA ratings: β = 0.15, SE = 0.24, z = 0.64, *p* = 0.524; encoding duration: β = −0.01, SE = 0.10, z = −0.14, *p* = 0.892) or in the incongruent condition (SoA ratings: β = −0.38, SE = 0.27, z = −1.38, *p* = 0.167; encoding duration: β = −0.04, SE = 0.10, z = −0.38, *p* = 0.701). These null effects should be interpreted cautiously, as restricted within-condition variability in SoA ratings likely limited the sensitivity of these analyses.

In summary, these results suggest that the congruency manipulation and the related changes in the SoA, but not encoding duration, were key factors influencing participants’ accuracy in the spatial memory task, for correctly recognized old images.

#### RTs in left/right response

As spatial accuracy was significantly affected by congruency and SoA ratings, RT analyses were restricted to trials with correct spatial location responses. In these models, none of the predictors were significant (model 1: congruency: β = −0.02, SE = 0.04, *t* = −0.55, *p* = 0.583; encoding duration: β < 0.01, SE = 0.04, *t* = 0.03, *p* = 0.974; model 2: SoA ratings: β = −0.02, SE = 0.04, *t* = −0.37, *p* = 0.708; encoding duration: β < 0.01, SE = 0.04, *t* = 0.03, *p* = 0.980).

### Participants believed they remembered congruent trials better

Immediately after completing the recognition session, participants reported their subjective beliefs about whether memory performance differed between congruent and incongruent trials. One-sample *t*-tests against the neutral midpoint of 50 (indicating no perceived impact of task conditions on memory) showed that participants subjectively believed they had better memory for images encoded in the congruent condition. This effect was significant for memory performance both in the item recognition task (M = 61.60, SD = 30.46), *t*(29) = 2.09, *p* = 0.046, Cohen’s *d* = 0.38, and in the spatial memory task (M = 65.63, SD = 27.35), *t*(29) = 3.13, *p* = 0.004, Cohen’s *d* = 0.57.

### Observed memory effects were not explained by encoding time, spatial bias, or guessing

During encoding, participants were instructed to avoid mechanical response patterns, e.g., choosing the same side in all trials or alternating left and right across trials without engaging in meaningful categorization. Inspection of response patterns verified compliance, and no participants showed systematic mechanical responding. A paired-samples *t* test showed that RTs for selecting the left or right box did not differ significantly between conditions (congruent: M = 1.18, SD = 0.45; incongruent: M = 1.17, SD = 0.45), *t*(29) = 0.21, *p* = 0.838, Cohen’s *d* = 0.04. This indicates that participants had comparable image encoding durations between conditions. Additionally, a paired-samples *t* test showed that there was no significant difference in the proportion of left-box choices between conditions (congruent: M = 0.51, SD = 0.16; incongruent: M = 0.53, SD = 0.17), *t*(29) = −0.80, *p* = 0.430, Cohen’s *d* = −0.15, showing that participants had similar left-right box choice patterns between conditions. Furthermore, one-sample *t*-tests against a chance level of 0.5 showed that the proportion of left-box choices did not differ significantly from chance in either condition (congruent: *t*(29) = 0.28, *p* = 0.780, Cohen’s *d* = 0.05; incongruent: *t*(29) = 0.98, *p* = 0.337, Cohen’s *d* = 0.18), indicating that participants selected the left and right boxes with approximately equal frequency. Because final image locations were determined by selected or unselected boxes depending on condition, the nearly equal distribution of left- and right-box choices in each condition confirms that there was no spatial (left/right) bias in final image locations, thereby ruling out a potential confound for spatial memory.

For the recognition session, a paired-samples *t* test showed that the overall hit rate (i.e., the proportion of “old” responses to old images; M = 0.67, SD = 0.16) was significantly higher than the false alarm rate (i.e., the proportion of “old” responses to new images; M = 0.14, SD = 0.12), *t*(29) = 16.35, *p* < 0.001, Cohen’s *d* = 2.99. Additionally, a one-sample *t* test against a chance level of 0.5 showed that the overall spatial accuracy for correctly recognized old images was significantly above chance (M = 0.59, SD = 0.12; *t*(29) = 4.18, *p* < 0.001, Cohen’s *d* = 0.76). These results indicate that participants were able to remember images and their locations and were not simply guessing. Lastly, separate one-sample *t*-tests showed that spatial accuracy in the congruent condition was significantly above the chance level of 0.5 (M = 0.69, SD = 0.15; *t*(29) = 6.86, *p* < 0.001, Cohen’s *d* = 1.25), whereas spatial accuracy in the incongruent condition did not differ significantly from chance (M = 0.50, SD = 0.21; *t*(29) = −0.12, *p* = 0.909, Cohen’s *d* = −0.02). This pattern suggests that spatial memory was selectively supported in the congruent condition. Notably, if participants had relied on their intended (chosen) location in the incongruent condition (rather than the actual final location, as instructed), accuracy would have fallen below chance. Instead, performance was at chance level, indicating that participants genuinely failed to remember the final location in the incongruent condition.

## Discussion

The present study examined whether the SoA experienced during encoding modulates subsequent EM for objects and their spatial locations. Our manipulation of SoA was effective, as participants reported higher SoA ratings in the congruent condition, where object movement matched their chosen keypress, compared to the incongruent condition. We report three main findings. (1) Congruent action-outcome mappings and the associated increase in SoA ratings were associated with faster responses in the item recognition task, without affecting recognition sensitivity. Most notably, (2) congruency and the accompanying increase in SoA ratings predicted better spatial memory accuracy. Control analyses showed that (3) these effects could not be explained by differences in encoding duration, spatial distribution bias, or pure guessing. Together, these findings suggest that SoA during encoding selectively strengthens memory for self-caused spatial information.

Stronger SoA over object displacement at encoding was associated with faster Old/New recognition responses, while recognition sensitivity remained unchanged. This effect was evident when RTs were predicted by action-outcome congruency as well as by trial-by-trial subjective SoA ratings, after controlling for encoding duration. In the present study, congruency and SoA ratings were conceptualized as indirect and direct indicators of SoA, respectively. Although congruency primarily manipulates action-outcome predictability rather than SoA per se, higher SoA ratings in the congruent relative to the incongruent condition support its validity as a proxy for the SoA component of action-effect contingency. Their effects on memory performance were therefore examined in parallel to provide converging evidence for SoA-related effects on EM. Importantly, since recognition sensitivity did not differ between conditions and the RT effect disappeared when analyses were restricted to correct trials, faster responses likely reflect facilitated decision processes or increased confidence at retrieval rather than stronger mnemonic evidence. This interpretation is consistent with participants’ subjective belief that they remembered objects better in the congruent condition. In contrast, recognition sensitivity was primarily predicted by encoding duration, consistent with well-established exposure-time effects on memory.^31^^,^^32^ Thus, SoA appears to influence retrieval-related decision processes rather than item memory quality per se.

The most robust effect of SoA emerged for spatial aspects of EM. Compared to the incongruent condition, the congruent condition led to higher accuracy in recalling object locations 1 h later. During encoding, participants intentionally memorized both the objects and their final locations. Congruent trials reinforced a coherent action-outcome relationship, whereas incongruent trials violated participants’ predictions about the action-outcome. Although prediction errors typically attract attention and engage cognitive resources,^33^^,^^34^ which might be expected to enhance memory in the incongruent condition, the opposite pattern emerged: spatial memory was better in the congruent condition. This indicates that the spatial memory advantage cannot be explained by increased attentional capture or cognitive effort. Instead, higher SoA ratings during encoding predicted higher accuracy in the spatial memory task, supporting the interpretation that agency experience itself facilitates the encoding of action-related spatial information. RTs in the spatial location judgements did not differ between conditions, likely due to the sequential recall procedure in which participants made the old/new decision before reporting location. Spatial information may have been retrieved during the initial recognition phase, rendering subsequent RTs less sensitive to SoA-related effects.

Importantly, the observed spatial memory advantage cannot be explained by differences in motor engagement or action voluntariness, since participants executed identical keypresses and freely chose their actions in all trials. Our manipulation specifically targeted action-outcome congruency, that is, whether the sensory outcome (object movement and final spatial location) was contingent on participants’ actions. Therefore, the observed memory effects are distinct from production effects,^35^^,^^36^^,^^37^ which arise from performing overt actions during encoding (e.g., reading aloud, typing) regardless of whether those actions causally determine the ensuing outcomes. Moreover, the effect of action-outcome congruency on memory cannot be reduced to a general compatibility or congruency effect. Previous work has demonstrated an action-object compatibility effect, whereby performing an action that is incompatible with an object’s affordances during encoding impairs subsequent memory (e.g., pantomiming a swinging-chopping action while encoding a corkscrew rather than a hammer^38^). In that paradigm, the action and the to-be-remembered object are presented concurrently, and congruency is defined by the match between the performed action and the object’s affordances. In contrast, in our task, the action precedes the outcome, and congruency is defined by the match between an action and its subsequent sensory consequence. Thus, the observed memory effect in our task is distinct from this previously reported action-object compatibility effect.^38^ Additionally, our SoA manipulation specifically concerns participants’ ability to control objects via motor actions, which distinguishes it from potential cue-outcome congruency effects in the absence of action. Such cue-outcome congruency effects on memory could be examined in future work using a passive observation version of the task.

The spatial memory advantage in congruent trials is unlikely to be driven by rule-based responding. During the location memory task, if participants had relied solely and intentionally on remembered categorization rules or intended object locations rather than memory for the object’s actual final location (contrary to task instructions), performance in the incongruent condition should have fallen below chance, as intended and actual locations were opposite. Instead, spatial accuracy in the incongruent condition was at chance, indicating genuine uncertainty about the object’s location. While it remains possible that participants occasionally remembered only the intended location and responded based on this during the location judgment, this tendency itself reflects an effect of SoA on memory representations. Incongruent trials involve a mismatch between intended and actual outcomes, which may introduce contextual or source confusion during encoding and retrieval. However, we argue that contextual encoding confusion should not be viewed as an alternative explanation that replaces SoA, but rather as a possible cognitive mechanism through which disrupted SoA affects memory representations. Participants were explicitly informed about the subsequent memory test and instructed to encode the final object locations regardless of congruency. Despite this intentional encoding goal, spatial memory accuracy was significantly lower in the incongruent than in the congruent condition. This pattern suggests that action-outcome incongruency affects memory encoding in ways that cannot be fully compensated for by intentional encoding strategies, consistent with the idea that mismatches between intended and actual outcomes (i.e., reduced SoA) impair binding of event features into a coherent EM. Additionally, the observed effect cannot be attributed to differences in encoding duration, as trial-by-trial image presentation time neither predicted subsequent spatial memory nor differed between conditions. Moreover, the duration available for encoding the object-location association was constant across trials: Spatial information became available only once the object started to move and was displayed for a fixed 1000 ms in all trials. Likewise, this effect cannot be explained by spatial bias in final image locations, as participants placed approximately half of the objects in the left box and half in the right box in both conditions. Finally, participants’ performance reflected genuine memory rather than pure guessing, as the overall hit rate exceeded the false alarm rate and the overall spatial accuracy was above chance.

Our findings extend prior work demonstrating SoA-related memory benefits, which have largely focused on immediate or short-term memory. Hon and Yeo^26^ showed that recognition memory for neutral words improved when participants experienced stronger SoA over the words’ movements on the screen, manipulated through spatial congruency and action-outcome delay, and later studies replicated this effect.^25^^,^^27^ Importantly, in these studies, memory was tested immediately after encoding, encoding was incidental, and memory was assessed solely using item recognition, capturing short-term memory processes rather than long-term EM. In contrast, the present study examined long-term EM using a 1-h retention interval, intentional encoding, and a spatial memory test in addition to item recognition. Our results revealed a selective enhancement of spatial rather than item memory. While these task differences may contribute to variability in observed effects, they likely reflect complementary expressions of the same underlying mechanism: Stronger SoA enhances memory for information directly linked to self-generated outcomes. In our task, the SoA manipulation concerned the object-location relationship rather than object identity, as participants viewed the objects before the congruency manipulation but experienced different action-outcome contingencies afterward. In the key experiments of previous studies,^25^^,^^26^^,^^27^ participants pressed a key to move a box either congruently or incongruently, and the to-be-remembered word appeared only after the box stopped moving. Thus, the word was the outcome of their action, albeit indirectly and delayed, since the box’s movement was the immediate consequence. This indirect contingency may account for the relatively modest and less replicable effects on item memory (see Tsuji and Imaizumi^28^ for a null result). Similarly, Schreiner and colleagues^27^ showed that among several determinants of SoA in their task, only spatial congruency modulated recognition performance in an immediate surprise test. This finding reinforces the idea that SoA preferentially facilitates memory for information that is directly contingent on one’s own actions. In our task, the outcome, i.e., the object’s location change, was the direct consequence of participants’ actions, thereby producing a strong SoA-related benefit on spatial memory.

Notably, our theoretical claim is not that SoA enhances memory for all self-caused action outcomes, but that SoA can facilitate memory when actions and their outcomes are informative, task-relevant, or novel. In everyday life, highly habitual events (e.g., switching on a light in one’s living room) are overlearned, low in novelty, and carry little informational value, and are therefore unlikely to be encoded episodically. In contrast, the events in our task involve novel stimuli and unpredictable outcomes, making them more likely to benefit from SoA-related prioritization during encoding. Importantly, the present findings are based on emotionally neutral stimuli and a relatively constrained manipulation of SoA (congruent versus incongruent). Even in the incongruent condition, participants retained some degree of control (e.g., they voluntarily chose which button to press). In light of evidence that events involving a profound loss of SoA (e.g., traumatic or violent experiences) may give rise to exceptionally strong and persistent memory traces, often with clinically relevant consequences (e.g., post-traumatic stress disorder^39^), the relationship between SoA and memory is likely context-dependent and non-linear. In highly salient or emotionally charged contexts, memory formation may be dominated by factors such as emotional arousal or valence,^40^ potentially attenuating or overriding the influence of SoA. Future work should employ more graded manipulations of SoA (e.g., full, partial, and no control) and also examine how SoA influences memory in different emotional contexts.

Two complementary mechanisms may underlie this effect. First, a stronger SoA may increase the self-relevance of an event, thereby promoting deeper encoding and more accessible retrieval. Information tagged as self-related reliably enjoys a memory advantage over information perceived as unrelated to the self, a phenomenon known as the self-reference effect.^41^^,^^42^^,^^43^ This advantage extends to objects only incidentally associated with the self. For example, Kim and Johnson^44^ found that objects arbitrarily assigned as “belonging to the self” were remembered better than those assigned to another person. Stampacchia and colleagues^45^ also showed that self-relevant memories ameliorated memory deficits in patients with fronto-parietal stroke. A heightened SoA similarly signals that an outcome is self-caused and therefore personally enacted, thereby engaging self-referential processes that enhance memory formation. Second, experiences accompanied by high SoA may be intrinsically rewarding, resulting in more durable episodic traces. Prior studies have shown that the emotional valence of action outcomes (e.g., rewarding or punishing sounds, monetary incentives) can modulate SoA,^46^^,^^47^ and emotional valence or arousal is well known to modulate memory.^5^ It is therefore possible that emotionally salient action outcomes influence SoA and memory in parallel. In the present paradigm, however, action-outcome congruency was used to manipulate SoA with affectively neutral outcomes (i.e., movement direction carried no explicit reward or punishment value). Accordingly, we interpret our findings as being consistent with the possibility that having control over environmental objects (i.e., SoA) itself may be rewarding and carry motivational value, even in the absence of explicit rewards, and thereby bias memory encoding in favor of high-SoA events. This interpretation is in line with previous evidence showing that actions associated with higher SoA are preferred and more readily executed,^11^^,^^48^ and that high action-effect contingency^49^ and perceived control^50^^,^^51^ engage reward-related regions such as the striatum. Although the present design cannot dissociate these mechanisms, both accounts converge on the idea that SoA strengthens event integration by increasing the personal and motivational significance of experience. Future neuroimaging work will be necessary to disentangle their neural bases.

In conclusion, congruency between actions and their consequences and the related SoA during encoding enhances EM, primarily for self-caused changes in the location of objects. This SoA-driven enhancement likely arises because self-generated, causally congruent events are more self-relevant and motivationally significant. Our findings highlight that SoA functions as a cognitive binding mechanism, integrating actions and their perceptual consequences into coherent episodic representations.

### Limitations of the study

Several limitations and future directions should be noted. First, SoA was assessed using explicit ratings, which may be influenced by introspective limits and demand characteristics. Future studies could combine explicit ratings with implicit measures of SoA (e.g., intentional binding or neural markers) and include control ratings (e.g., emotional arousal). In addition, SoA ratings showed a skewed distribution, with high ratings in the congruent condition and low ratings in the incongruent condition. As action-outcome congruency was explicitly designed to manipulate subjective SoA in the present paradigm, SoA and congruency are necessarily intertwined. Future work that orthogonally manipulates action-outcome contingency and perceived control will be required to more fully dissociate their respective contributions to memory. Relatedly, the present design cannot fully disentangle contextual encoding confusion from SoA-related effects; future studies could orthogonally manipulate intention-outcome conflict and perceived SoA to better separate their respective influences on memory. Second, the current SoA manipulation relied on a simple mapping between keypresses and object movements on a screen, a well-established but relatively indirect approach. More immersive paradigms using virtual reality or motion tracking could allow for continuous, naturalistic manipulations of SoA and tighter coupling between actions and their effects linked to bodily agency and its impact on EM, as recently reported.^52^^,^^53^^,^^54^ Third, memory was tested only after a 1-h delay. Future studies could include multiple retention intervals, including overnight consolidation, to examine how the SoA-related memory advantage evolves over time and to clarify whether SoA influences encoding, consolidation, and/or retrieval. Finally, although biological sex was recorded, the sample size was relatively small and unbalanced (20 females, 10 males), and the study was not designed or powered to examine sex-related differences. Gender identity was not collected. This may limit the generalizability of the findings. Future studies with larger and more balanced samples should explore potential sex- and gender-related effects.

## Resource availability

### Lead contact

Requests for further information and resources should be directed to and will be fulfilled by the lead contact, Qiaoyue Ren (qiaoyue.ren@epfl.ch).

### Materials availability

This study did not generate new unique reagents.

### Data and code availability

• Raw and preprocessed behavioral data have been deposited at Open Science Framework and are publicly available as of the date of publication at https://doi.org/10.17605/OSF.IO/ZTNS2.
• All original code has been deposited at Open Science Framework and is publicly available at https://doi.org/10.17605/OSF.IO/ZTNS2 as of the date of publication.
• Any additional information required to reanalyze the data reported in this paper is available from the lead contact upon request.

## Acknowledgments

This work was supported by the Swiss National Science Foundation (SNSF) under an Grant [TMPFP3_234527] (Swiss Postdoctoral Fellowship) to Qiaoyue Ren and by support from the EMPIRIS foundation, the Synapsis foundation, by two generous donors advised by CARIGEST SA (Fondazione Teofilo Rossi di Montelera e di Premuda and a second one wishing to remain anonymous) to Olaf Blanke.

## Author contributions

Conceptualization, Q.R., B.H., S.S.-B., and O.B.; Methodology, Q.R., B.H., N.H.M, S.S., S.S.-B., and O.B.; Investigation, Q.R.; Writing—original draft, Q.R.; Writing—review & editing, B.H., N.H.M, S.S., S.S.-B., and O.B.; Funding acquisition, Q.R. and O.B.; Resources, O.B.; Supervision, O.B.

## Declaration of interests

The authors declare no competing interests.

## STAR★Methods

### Key resources table

**Table:** 

REAGENT or RESOURCE	SOURCE	IDENTIFIER
**Deposited data**		

Raw and analyzed data	This paper	https://doi.org/10.17605/OSF.IO/ZTNS2

**Software and algorithms**		

R version 4.5.0	https://www.r-project.org/	https://www.r-project.org/
RStudio version 2025.09.0	https://posit.co/download/rstudio-desktop/	https://posit.co/download/rstudio-desktop/
PsychoPy version 2024.1.5	https://www.psychopy.org/	https://www.psychopy.org/
JASP version 0.95.4.0	https://jasp-stats.org/	https://jasp-stats.org/

### Experimental model and study participant details

#### Participants

Thirty healthy human adults (*N* = 30; 20 females, 10 males; mean age: 26.73 ± 4.53 years; range: 18–37 years) took part in this study. All participants were right-handed and had normal or corrected-to-normal vision, no color blindness, no history of psychiatric or neurological disorders, and were not taking any medication. Information regarding participants’ gender, ancestry, race, ethnicity, and socioeconomic status was not collected and is therefore not available. The experiment used a within-subject design in which all participants completed all task conditions. *A priori* power analysis using G∗Power^55^ indicated that a minimum of 28 participants would be required to detect a within-subject effect of the SoA manipulation on memory (Cohen’s *d* = 0.55, estimated based on effect sizes reported in related studies^19^^,^^26^^,^^27^), with α = 0.05 and power = 0.80. Written informed consent was obtained from all participants, and they were compensated 20 CHF per hour. The study was approved by the local ethical committee (Cantonal Ethical Committee of Geneva: 2015-00092) and conducted in accordance with the Declaration of Helsinki.

### Method details

#### Materials and apparatus

Ninety-six colored images of objects were selected from the validated Snodgrass and Vanderwart object pictorial set.^56^^,^^57^ All objects depicted were emotionally neutral, common, and non-living. Familiarity ratings (*F*(2,58) = 0.27, *p* = 0.764, *η*_*p*_^2^ = 0.01), complexity ratings (*F*(2,58) = 2.64, *p* = 0.080, *η*_*p*_^2^ = 0.08), and the proportion of manmade versus non-manmade objects (*F*(2,58) = 2.37, *p* = 0.103, *η*_*p*_^2^ = 0.08) were comparable across the congruent, incongruent, and new conditions in the present study. The images were presented at a size of 300 × 300 pixels (width × height) on a gray background, displayed on a 24-inch monitor (refresh rate: 60 Hz; resolution: 1920 × 1080 pixels) at a viewing distance of approximately 65 cm. The experiment was programmed and run using PsychoPy (version 2024.1.5).^58^

#### Design and procedure

The experiment consisted of two sessions: a memory encoding session and a recognition session, separated by a 1-h break.

On each trial of the encoding session (see Figure 1A), participants viewed an image of an object presented at the center of the screen, flanked by two black boxes (300 × 300 pixels) positioned on the left and right, centered at 25% and 75% of the screen width, respectively. They were instructed to sort each object into the left or right box by pressing the corresponding arrow key (left or right) with the index or middle finger of their right hand, respectively, using self-chosen categorization rule. The specific rule was unconstrained and could vary across trials, as it was not of theoretical interest and served only to ensure active engagement with each stimulus. If no response was made within 3 s, a “hurry up” message would appear below the image.

Following the keypress, the object moved either to the left or right box. This movement lasted 500 ms, and the object then remained in its final location (i.e., inside the box; visible) for an additional 500 ms. Previous studies have shown that participants experience a stronger SoA when their actions produce predictable outcomes and a diminished SoA when outcomes are unexpected.^29^^,^^30^ Accordingly, we implemented two trial types: congruent and incongruent trials. In congruent trials, the object moved in the same direction as the participant’s chosen keypress (e.g., pressing the right arrow key moved the object to the right box). In incongruent trials, the object moved in the opposite direction (e.g., pressing the right arrow key moved the object to the left box).

At the end of each trial, a visual analog scale appeared on the screen. Participants rated their SoA by answering the question, “In the previous trial, how much did you feel your keypress controlled the object’s relocation?” on a scale from 0 (no control) to 100 (full control) in increments of 1. They adjusted the rating using the “G” and “H” keys on the keyboard to decrease or increase the value, respectively, and confirmed their rating with the “Space” key, using their left hand. The starting position of the cursor on the scale was randomized on each trial. A central fixation cross was presented for 1000–2000 ms between trials.

Participants were informed before the encoding session that their memory for the objects and their final locations (left or right) would be tested 1 h later. They were therefore encouraged to intentionally encode both the images and their spatial locations, with the aim of enhancing overall memory performance and reducing potential attentional differences between conditions. Additionally, to minimize spatial distribution bias that could confound later memory performance, participants were instructed to distribute the objects approximately equally between the left and right boxes. They were also asked to avoid mechanically selecting the same side across multiple trials (e.g., pressing left for the first half of the task and right for the second half), or simply alternating between sides (e.g., left, right, left, right), without engaging in meaningful categorization. Instead, they were encouraged to make thoughtful placement decisions, thereby promoting deeper memory encoding. Additionally, participants were instructed that their keypress should reflect the location to which they intended to move the object, and not to attempt to counteract the manipulation by pressing the opposite key in anticipation of incongruent trials. The specific categorization rules used during the task were not formally recorded. However, post-task oral reports indicated that participants employed a variety of rules and frequently adjusted them across trials, which is unsurprising given that the full stimulus set was not known in advance and spanned a wide range of categories.

The encoding session included 4 blocks, each containing 12 trials (6 congruent and 6 incongruent). Trial order was pseudo-randomized for each participant, with the constraint that no more than three consecutive trials belonged to the same condition. Each trial displayed a unique image, with 48 images randomly selected from a pool of 98. Participants were given a 20-s break after each block. The entire encoding session lasted approximately 15 min. Participants then took a 1-h break within the laboratory area before the memory test. Participants could engage in non-demanding activities (e.g., walking, light reading) but were asked to avoid memory-demanding tasks, and electronic devices were not allowed.

In the recognition session (see Figure 1B), participants were shown 96 images: the 48 “old” images from the encoding session and 48 “new” images that had not been presented before. Each image was displayed once in a random order. For each image, participants first indicated whether they had seen it during the encoding session by pressing a key with their right hand (left or right arrow key corresponding to “Old” or “New,” respectively). If they responded “Old,” they were then asked to recall whether the object had moved to the left or right box during encoding (left or right arrow key corresponding to “Left” or “Right,” respectively). If they responded ‘New,’ they were instructed to indicate whether they would like to place the object on the left or right, with both choices considered correct. In other words, participants answered two questions for each image, regardless of whether it was considered old or new. This design prevented participants from reducing task demands by strategically selecting “New” to avoid the follow-up spatial location question, which might have occurred if only “Old” responses had triggered the second question. A central fixation cross appeared for 1000–2000 ms between trials. Participants were instructed to respond as quickly and accurately as possible. Immediately following each keypress, the selected option on the screen changed color to confirm their choice. This visual change served only as response confirmation and did not provide any information about response accuracy.

The recognition session included 4 blocks, each containing 24 trials: 6 old images from the congruent condition, 6 from the incongruent condition, and 12 new images. Trial order was pseudo-randomized for each participant, with the constraint that no more than three consecutive trials came from the same condition. The entire recognition session lasted approximately 15 min.

Immediately after completing the recognition session, participants rated their subjective feelings about their memory performance in the congruent and incongruent conditions, using two visual analogue scales. The first statement was: “I am more likely to recognize the object if it moved to the box I chose in the previous session.” The second was: “I am more likely to recall the location of the object if it moved to the box I chose in the previous session.” For both statements, participants responded on a scale from 0 to 100, where 0 indicated “strongly disagree” (i.e., perceived better memory for objects in the incongruent condition), 100 indicated “strongly agree” (i.e., perceived better memory in the congruent condition), and 50 indicated no perceived difference between the two conditions.

To familiarize participants with the procedure, they completed 8 practice trials (4 congruent and 4 incongruent) before the encoding session and 2 practice trials before the recognition session. All practice trials used the same image, which was not included in the main task.

### Quantification and statistical analysis

#### SoA ratings

To confirm the effectiveness of the manipulation of the SoA, we compared the subjective SoA ratings between the congruent and incongruent conditions during the encoding session using a paired-samples *t* test.

#### Memory performance

Participants’ memory performance was assessed through two measures: (1) Old/New recognition response, reflecting item memory, and (2) subsequent Left/Right response for recognized items, reflecting spatial memory. Generalized linear mixed-effects models (GLMMs) were used for binary outcome variables, whereas linear mixed-effects models (LMMs) were used for continuous outcome variables.

To assess item recognition while accounting for response bias, analyses of the Old/New response included both old and new test items, rather than being restricted to old items alone. Including both item types allows recognition performance to be decomposed into response bias (i.e., the general tendency to respond “old”) and recognition sensitivity (i.e., old-new discriminability), consistent with the logic of signal detection theory.^59^ Specifically, the Old/New response (1 = “old”, 0 = “new”) was analyzed using GLMMs with a binomial distribution and logit link function. The predictor *is_old* (1 = old item, 0 = new item) distinguished signal from noise, such that the model intercept indexed overall response bias, whereas the effect of *is_old* indexed overall recognition sensitivity.

Two GLMMs were specified to separately examine the effects of (1) objective task conditions (action-outcome congruency: congruent vs. incongruent) and (2) subjective agency experience (SoA ratings) on recognition sensitivity. Congruency and SoA ratings were treated as parallel indicators of SoA and modeled in separate analyses because SoA ratings are inherently dependent on the congruency manipulation; including both predictors simultaneously would induce substantial collinearity and complicate interpretation.

In the congruency model, congruency was coded as +0.5 (congruent) and −0.5 (incongruent), with new items coded as 0. Congruency and z-scored encoding duration (RTs for selecting the left or right box at encoding) were included via their interactions with *is_old*, such that these predictors specifically tested whether experimental manipulations modulated recognition sensitivity (i.e., old-new discriminability). In the SoA model, z-scored SoA ratings and z-scored encoding duration were similarly included via interactions with *is_old*. Notably, encoding duration was included to control for potential confounding effects of encoding time on subsequent memory performance. Both models included random intercepts for participant and image to account for subject- and item-level variability. RTs for the Old/New response were analyzed using LMMs with the same fixed and random effects structure. To reduce the influence of extreme values and improve adherence to model assumptions, RT outliers (>3 SDs above each participant’s mean) were excluded. This resulted in the removal of an average of 1.93 trials per participant (SD = 1.05; range = 0–5), corresponding to 2.01% of trials.

For the Left/Right response, the same modeling approach was applied. Accuracy was analyzed using GLMMs, restricted to trials with correct “Old” responses to the Old/New question. RTs were analyzed using corresponding LMMs, limited to trials with correct location responses. Excluding RT outliers (>3 SDs above each participant’s mean) resulted in the removal of an average of 0.43 trials per participant (SD = 0.50; range = 0–1), corresponding to 1.31% of trials.

Additionally, participants’ subjective ratings of perceived differences in memory performance between congruent and incongruent trials were analyzed using one-sample *t*-tests against the neutral midpoint of 50 (indicating no perceived impact of task conditions on memory).

#### Control analyses

Several control analyses were conducted to rule out potential confounding factors.

For data from the encoding session, to assess whether images were presented for a comparable duration across congruent and incongruent conditions; that is, whether participants had similar encoding time between conditions, we compared the RTs for selecting the left or right box using a paired-samples *t* test. Additionally, to ensure there was no systematic left-right response bias, we compared the proportion of left-box choices between the two conditions using a paired-samples *t* test. Furthermore, one-sample *t*-tests against a chance level of 0.5 were used to assess whether the proportion of left-box choices in each condition did not differ from chance, i.e., whether participants selected the left and right boxes approximately equally.

For data from the recognition session, to assess whether participants’ Old/New responses reflected true item memory rather than chance-level responding, we compared the overall hit rate across conditions (i.e., the proportion of “Old” responses to old images) with the false alarm rate (i.e., the proportion of “Old” responses to new images) using a paired-samples *t* test. To evaluate spatial memory, one-sample *t*-tests against a chance level of 0.5 were conducted on Left/Right response accuracy for correctly recognized old images, both overall and separately for the congruent and incongruent conditions.

All analyses were conducted using JASP (version 0.95.4.0),^60^ R (version 4.5.0) and RStudio (version 2025.09.0). We report *t*-values, *p*-values, and Cohen’s *d* for paired-samples and one-sample *t*-tests. All mixed-effects models were fitted using maximum likelihood estimation. For fixed effects, we report parameter estimates (β), standard errors (SE), *z*-values (for GLMMs) or *t*-values (for LMMs), and *p*-values. Effect sizes were reported as Cohen’s *d* for paired-samples and one-sample *t*-tests, partial eta squared (*η*_*p*_^2^) for repeated-measures ANOVAs, and standardized regression coefficients (β) for GLMMs and LMMs. All tests were two-tailed, and results were considered statistically significant when *p* < 0.05. The sample size (N) refers to the number of participants. Descriptive data are reported as mean ± standard deviation (SD). Detailed statistical results, including test statistics and exact *p*-values, are reported in the results section and Table S1.
