# Supplementary material for: Sense of agency strengthens memory for self-caused spatial information
Source: iScience. 2026 Mar 16;29(4):115382. doi: 10.1016/j.isci.2026.115382 (PMC13081049; doi:10.1016/j.isci.2026.115382)
Supplement: Document S1. Table S1 [file mmc1.pdf]

iScience, Volume *29*

Supplemental information

Sense of agency strengthens memory

for self-caused spatial information

Qiaoyue Ren, Bruno Herbelin, Nathalie H. Meyer, Sara Stampacchia, Simone Schütz-Bosbach, and Olaf Blanke

Table S1. Memory performance, related to STAR Methods

	**Congruent**	**Incongruent**	**New**
Old/New Response			
Hit rates	0.66 ± 0.16	0.67 ± 0.19	–
False alarm rates	–	–	0.14 ± 0.12
RTs in all trials	1.70 ± 0.49	1.81 ± 0.57	–
RTs in correct trials	1.66 ± 0.51	1.73 ± 0.56	–
Left/Right Response			
Accuracy	0.69 ± 0.15	0.50 ± 0.21	–
RTs in correct trials	1.17 ± 0.66	1.22 ± 0.64	–

Note. Hit rates refer to the proportion of “old” responses to old items; false alarm rates refer to the proportion of “old” responses to new items; reaction times (RTs) are reported in seconds.
